# Round the Hospitals

**Published:** 1920-08-28

**Authors:** 


### ROUND THE HOSPITALS.

The annual report of the work of the Samaritan Fund at St. Thomas's is one of the most educative documents we know. Under the able guidance of the matron and the inspiration of the Secretary and Chief Almoner, Miss A. E. Cummins, admirable work of a kind which imparts new vigour to the roots of a nation is being carried on, patiently, economically, successfully. The report should be in the hands of all who labour at social reconstruction. The Samaritan Fund now embraces a wide range of operations for the good of patients and their families. Its convalescent branch sent away 861

**ROUND THE HOSPITALS—(*continued*).**

patients, for periods varying from three weeks to nine months. In all, 2,579 patients, in and out, were provided with surgical instruments, only a fractional sum of the cost falling on the Samaritan Fund, for the rest was repaid in instalments. The casualty and out-patient departments work in close touch with the Samaritan Fund, and numerous welfare workers, baby clinics, and Government departments depend on it for advice and help. The speech clinic is something of a new departure and deals with widespread and important defects which have hitherto had scant attention paid to them. Immense difficulties fall on the female side of the venereal department, which is developing in ways undreamed of ten years ago. And the maternity department, baby centre, and day nursery deserve a page to themselves.

---

The news that the London Centre of the College of Nursing has secured premises with ample bedroom accommodation has caused great satisfaction in the ranks of members outside the Metropolis. For great inconvenience is caused to members travelling up to attend meetings and conferences by the scarcity of sleeping quarters at present. The new residential club will be close to the present offices of the College, and its restaurant, which is to be run on high-class lines, with due regard to members' purses, will be a great boon to the enormous body of nurses whose business centres within a mile radius of that quarter.

---

An effort is being made to revive the extremely profitable "Our Day" celebrations on behalf of the Red Cross this year. A campaign will be held from October 9 to 13, and energetic steps are being taken to ensure that the whole community participates in the rally. The needs of the hospitals have never been so insistent before, and the twin societies of the Red Cross and St. John are vowed to come to the rescue. The responsibilities which they are assuming under their new programme of work are vastly more onerous than those of war-time, because they have to be taken over in perpetuity instead of for a limited period. Much has already been accomplished for the benefit of the civilian sick. In particular an adequate motor ambulance has been installed in many parts of the kingdom. The substantial sum of a quarter of a million has been distributed, through King Edward's Hospital Fund, to the London hospitals, and a great extension of grants-in-aid to local hospitals is forecasted. Working parties for the supply of necessaries to the hospitals have been organised in many districts, and in one or two places there are signs that voluntary aid will be made available to help nurses in emergency. But this latter feature of Red Cross work still hangs unaccountably.

---

We are disposed to believe that considerable success would attend a well-organised attempt on the part of the hospitals to combine with the "Our Day" movement, and use the week which is set apart for interesting the public in the needs of the sick to make known their pressing need for nurses. To form a nursing bureau in charge of a nursing sister in uniform in every place which is celebrating "Our Day" should not be difficult. Information should be available as to all kinds of nursing. Opportunities should be given by the matrons of institutions, voluntary and municipal, for visitors to see the wards and nurses' home. Leaflets should be prepared setting forth the facts relating to pay, length of training, duty and off-duty hours, and the various branches of the profession to which training affords admission; and in every cinema theatre during the week a short film giving pictures of hospital life from the moment the probationer enters should be shown. To improve the nurses' lot it is essential to have a better supply of probationers, and the few hospitals which do not suffer from a lack of candidates ought to help their comrades in the hour of need and use their influence to awaken a vocation in the earnest-minded women required in the profession.

---

A great step has been taken towards the institution of a one-portal examination for candidates for training. The West Ham Board of Guardians, in their difficulties about procuring probationers, have consulted the College of Nursing, as well as the Ministry of Health. The College suggested that an entrance examination should be held, with the view of attracting a higher class of probationers, and it appears that the Ministry of Health regards the suggestion with favour, so that further developments may shortly be expected. It is not anticipated that women would enter for such examinations in sufficient numbers to fill all the vacancies. Indeed, it is rather with the hope of attracting educated women to training-schools able to offer rather special educational advantages that this examination would be instituted. But it is well worth a trial, and, we believe, would exercise a bracing influence, not only on the entrants, but on the schools which applied for the successful condidates.

---

The only road to attract women to the profession in large numbers is that of forming preliminary training-centres in different parts of London and the provinces. We rejoice to hear that the College of Nursing is taking the lead in inducing Boards of Guardians to co-operate with them to this end. There are at present nearly 50,000 women on the unemployed register. It may be reckoned that there are at least as many unemployed who have not registered as such. It is not conceivable that among these there should not be a large proportion of potential nurses. What is needed is short courses of instruction developed in connection with women's colleges, to open up to likely candidates the enormous attractiveness of nursing. That it has an irresistible magnetic draw for the right kind of woman has been proved over and over again. But the girls of the present day have been positively scared away

**ROUND THE HOSPITALS**—(*continued*).

from nursing. They have no conception of the substantial material advantages offered by the training-schools, of the extent to which they help to enlarge individual capacities, and, least of all, the deep inner joys nursing brings within reach. The only way to bring them to choose nursing for their career is to afford them short courses of preliminary instruction, and if these could embrace a glimpse of service in the wards so much the better.

It would appear that few branches of the profession are more profitable than that of the tuberculosis nurse. The present salaries of the Lambeth tuberculosis nurses is £316 a year, including war bonus. There is a strong feeling in the borough council, as we mentioned in our issue of the 14th inst., that they ought to be placed on exactly the same footing as the lady health visitors and sanitary inspectors in respect of salary, and should this be done the salaries would range from a minimum of £350 to a maximum of £450. Having due regard to the arduous character of the work and the very exact skill and precautions necessary to be observed in effectively carrying out the delicate duties of the tuberculosis visitor, none would contend the salary is excessive. If the disease is to be stamped out a great accession of trained nurses in this department of preventive work is indicated, and there could be no more profitable investment for any municipality.

One of the largest Poor-Law training-schools in Ireland is that at Belfast, and the recent prize-giving for nurses at this institution testified to the fact that the Guardians are doing their best to make it thoroughly efficient. The event synchronised with the advent of a new lady superintendent, Miss Campbell, who received a cordial welcome from the Board on her first appearance. The gold medallist was Nurse Elizabeth McGimpsey; silver medallist, Nurse Emily Farr; bronze medallist, Nurse Harriet M'Kernan. The medals were instituted six years ago, and are given by individual Guardians. The high character of the Belfast Infirmary is attested by the honourable positions held by many of its nurses in the United Kingdom and in the Colonies, while in their war services they won many distinctions.

The Lincoln Guardians have made no reply to the complaints of the superintendent, charge nurses, and probationers who have recently resigned. At the last meeting of the Guardians it was contended that the nursing staff did not wait for the Committee's report of the investigation into their grievance, and that nothing was stated as to why they were leaving. It seems highly improbable that the nurses would have taken this serious step had they been assured of immediate investigation into their grievances, and a determination on the part of the Guardians to alleviate the avoidable discomforts from which they allege themselves to be suffering. We understand the main complaint to have been that no attention whatever was paid to the representations of the staff. One of the Guardians pointed out that quite £50 had been spent in advertising recently the three vacancies, presumably for charge nurses, and that they had been compelled to accept the first three who presented themselves.

A very temperate letter from the Rev. John Davies, Rector of Vaynor, a member of the Merthyr Board of Guardians, appears in the local press, pressing for further consideration of the case of Sister Elvina Davies, who, it will be remembered, after seventeen years' faithful service at the Merthyr Tydfil Infirmary, was lately requested by the Guardians to resign. He suggests that by looking into the matter carefully something may be found which can account for "the recent bit of unpleasantness" at the institution, and remarks that to exercise discipline, either by a nurse or by anyone else, often requires "what is known as preventive grace, which some of us are only blessed with in small quantities." Seventeen years is the best part of a nurse's life, and a little friendly consideration would seem not uncalled for, in view of the fact that the conditions under which Sister Davies put in this honourable term of service were not of a particularly exhilarating kind.

The unaccountable neglect shown by the public to the wounded men still in hospital has turned out to be due not to apathy, but to lack of organisation. Inspiring letters to the Press from Lady Astor and others have had their effect, not in producing a volume of isolated offers of entertainment—in this respect general disappointment was caused—but in stimulating the formation of a Society to make plans for cheering the sick and wounded still under treatment. The organiser is Miss Maria Cunningham, and under the excellent name of the "Not Forgotten Association" she is rapidly attracting members to offer their services. Lady Haig, Lady Beatty, and Lady Trenchard have become President and Vice-Presidents. Large parties, numbering some hundreds, have already been entertained from Millbank, Roehampton, and Ducane Road Hospitals at private houses, and an active programme for the future is being arranged. Offers of help and gifts of money should be sent to the Secretary at 86 Ladbroke Road, W. 11. The new Society promises continuous and effectual steps towards lessening the monotony of hospital life for the heroes.

Lord Haig is ceaseless in his efforts to procure a satisfactory future for the officers and men who lost their chances in civil life by reason of their military service, and has succeeded in keeping their cause well before the public, which is the only road to secure them justice. Few persons realise the extent to which nursing sisters also have incurred financial loss and exclusion from the higher ranks of

**ROUND THE HOSPITALS**—(*continued*).

their profession through the same cause. The case of matrons of military and voluntary hospitals during the war is particularly hard. Many of these women were at the top of the tree and renounced highly honourable and lucrative posts in order to undertake the difficult task of organising the nursing departments of institutions for the wounded in all parts of Great Britain and abroad. They now find themselves stranded. The war cost them their former posts, and their age, with few exceptions, excludes them from civil appointments suitable to their capacity, for which there is the keenest possible competition. These war sisters and matrons have the first claim in our judgment to appointments under the Ministries of Health and Pensions. They should also receive more consideration than they do at the hands of the managers of voluntary and municipal institutions. Other things being equal, they ought to be given the preference in filling advantageous posts, and their period of military service might well be struck off in reckoning the age limit.

---

The average time on duty for day nurses in New York averages eleven hours twenty minutes, and for night nurses eleven hours fifty minutes. This information has been obtained by the Department of Health in New York City by means of a questionnaire circulated to seventy-two hospitals, containing in all 17,494 beds. The replies to the seventy-one questions asked are given in the *Trained Nurse* for August and present many interesting features. For instance, the average number of nurses in a room is two; the average age of admission of nurses in training is twenty-four; the average length of training is twenty-nine months, twenty-six days; the average time off-duty a day is two hours, twenty-two minutes. In nineteen hospitals the nurse's off-duty time can be taken away for infraction of rules. Two of the training-schools admit that the patients' and nurses' laundry is done together, a very flagrant neglect of the most elementary sanitary precaution. In eight hospitals a poorer quality of food is served to the nurses than that provided for the doctors. Social intercourse with the resident medical staff is prohibited in about one-half of the training-schools.

---

One of the Lewisham Guardians has informed his colleagues that he was witness recently of a deplorable waste going on at the hospital, where on inspection he found large quantities of bread and joints of meat in the waste-bins. After the meeting other Guardians examined the bins and found bread in them. The official explanation bears out our contention against the senseless allowancing system in vogue under the Poor Law. The bread, it was stated, had been left by patients, and could not be dealt with in any other way. Surely, with the quartern loaf standing at its present price, and a near prospect of a rise, the Guardians might take example from the frugal voluntary hospitals and let the patients have what bread they want, instead of weighing out to them what the authorities imagine they ought to eat. To check the crime of throwing good food into waste-bins no regulations suffice. The only effective method is to engage a housekeeper or steward who is an early riser and will personally supervise the emptying of the bins.

---

Widespread disappointment has been caused by Dr. Addison's statement in the House of Commons that the Third London General Hospital cannot be retained as a civilian hospital. The London County Council acquired the site from the Royal Patriotic Fund Corporation, and hold it on trust for an open space, and there is strong local opposition to its use in any other way. At the same time its use as a healing centre would, in the judgment of many residents, have outbalanced its value as a very small "lung" of Outer London, and one cannot hear without keen regret of the dismantling of this finely organised unit and the dispersion of the nursing staff.

---

Public Health nursing is making very rapid strides in America. The number has risen from 5,332 in the period preceding the declaration of war in that country to 8,770 on the first day of the current year. The best authorities reckon that at least 50,000 public health nurses are required to cover the most glaring needs of a population stretching from the Atlantic to the Pacific and from Florida to Alaska. We learn from the *Public Health Nurse* that one of the most isolated nurses in the world is working at a mission in Alaska, 100 miles from the nearest post office, at a place called "Chicken." She finds tuberculosis and syphilis are the great scourges of the Indians among whom she lives. The only means of transportation is by dog team. The future of civilisation is plainly seen to be bound up with the ministrations of health nurses, and the expression has a better sound than "sick nurses," though there may be little difference between the two.

---

Twelve years ago the Council of the Royal Sanitary Institute established an examination for health visitors and school nurses, and it has continued to keep pace with the steadily improving standard in this important branch of work. Pending further developments in the qualifications required of health visitors and child welfare workers, the Institute is continuing its course of training and examinations on the lines previously approved by the Local Government Board, and the autumn course will begin on September 24. The fee for the complete course of lectures and demonstrations is £5 5s., and an examination will be held at the close under regulations to be ascertained from the Secretary to the Royal Sanitary Institute, 90 Buckingham Palace Road, London, S.W. 1.

